# Relationships between aquatic vegetation and water turbidity: A field survey across seasons and spatial scales

**DOI:** 10.1371/journal.pone.0181419

**Published:** 2017-08-30

**Authors:** Åsa N. Austin, Joakim P. Hansen, Serena Donadi, Johan S. Eklöf

**Affiliations:** 1 Department of Ecology, Environment and Plant Sciences, Stockholm University, Stockholm, Sweden; 2 Baltic Sea Centre, Stockholm University, Stockholm, Sweden; University of Siena, ITALY

## Abstract

Field surveys often show that high water turbidity limits cover of aquatic vegetation, while many small-scale experiments show that vegetation can reduce turbidity by decreasing water flow, stabilizing sediments, and competing with phytoplankton for nutrients. Here we bridged these two views by exploring the direction and strength of causal relationships between aquatic vegetation and turbidity across seasons (spring and late summer) and spatial scales (local and regional), using causal modeling based on data from a field survey along the central Swedish Baltic Sea coast. The two best-fitting regional-scale models both suggested that in spring, high cover of vegetation reduces water turbidity. In summer, the relationships differed between the two models; in the first model high vegetation cover reduced turbidity; while in the second model reduction of summer turbidity by high vegetation cover in spring had a positive effect on summer vegetation which suggests a positive feedback of vegetation on itself. Nitrogen load had a positive effect on turbidity in both seasons, which was comparable in strength to the effect of vegetation on turbidity. To assess whether the effect of vegetation was primarily caused by sediment stabilization or a reduction of phytoplankton, we also tested models where turbidity was replaced by phytoplankton fluorescence or sediment-driven turbidity. The best-fitting regional-scale models suggested that high sediment-driven turbidity in spring reduces vegetation cover in summer, which in turn has a negative effect on sediment-driven turbidity in summer, indicating a potential positive feedback of sediment-driven turbidity on itself. Using data at the local scale, few relationships were significant, likely due to the influence of unmeasured variables and/or spatial heterogeneity. In summary, causal modeling based on data from a large-scale field survey suggested that aquatic vegetation can reduce turbidity at regional scales, and that high vegetation cover vs. high sediment-driven turbidity may represent two self-enhancing, alternative states of shallow bay ecosystems.

## Introduction

Aquatic vegetation (coarsely structured macroalgae and vascular plants) are foundation organisms in shallow coastal areas, and sustain multiple ecosystem services including fish production [1, 2], sediment stabilization [3] and nutrient filtering [4]. Many large-scale field surveys suggest the distribution of aquatic vegetation is limited by high water turbidity [5–10], which in coastal ecosystems is primarily influenced by runoff from land that brings dissolved nutrients and suspended particles [11, 12]. Suspended particles increase turbidity by absorbing light, while nutrients increase the growth of phytoplankton, which also absorbs light and thereby increases turbidity [13]. In addition, particles can re-suspend from the seafloor through physical disturbance, such as wave action, water currents [13, 14], and boat-mediated wake and currents [15].

Many small-scale experiments show that aquatic vegetation can also reduce turbidity by i) stabilizing the seabed, thereby decreasing sediment resuspension [3], ii) decreasing water movement, leading to increased sedimentation [16], and iii) reducing the growth of phytoplankton, through competition for nutrients [4] and/or by releasing allelochemical substances [17]. Using causal modeling based on field survey data, van der Heide et al. [18] showed that the seagrass *Zostera marina* can locally increase light penetration, partly by taking up nitrogen, and partly by stabilizing sediments. These results indicate that the regulating effect of aquatic vegetation on turbidity may have been underestimated in many field surveys, due to a lack of proper statistical tools.

The effect of organisms on abiotic conditions and, therefore, the provisioning of ecosystem services like good water quality is likely to vary over time [19], due to seasonal changes in abundance and distribution of organisms. For example, the ability of aquatic plants to attenuate waves [20], regulate nutrients [21] and absorb CO_2_ [22] is strongest in summer, when plant cover and biomass peak. Additionally, disturbance or altered environmental conditions can have stronger effect on an ecosystem during certain seasons, i.e. when they occur during the reproduction period or the early life stages of organisms. For example, plants with floating leaves, e.g. *Nymphaea*, can grow well in turbid waters, but clear water is needed early in the growing season for new seedlings to establish [23]. Consequently, accounting for seasonal variability is central when assessing relationships between organisms and ecosystem services.

Spatial scale is also an understudied but important factor that is likely to influence the effect of organisms on their environment, including ecosystem services. A recent study based on data from a global field survey suggests that organism-environment relationships can vary in strength and even direction with spatial scales, and that simple bivariate plotting of net relationships may obscure actual relationships [24]. Theory and empirical studies show that species distributions are generally structured by abiotic factors at regional scales, while the effects of organisms on their environment are typically more local [25]. However, the effect of biota might have been underestimated in many studies, due to the predominant use of statistical methods that explore net relationships, while organism-environment interactions consist of networks of direct and indirect effects [24, 26]. One of the methods that can tease apart direct and indirect interactions is structural equation modeling (SEM) [27, 28]. In the best of worlds, cause-effect relationships should be assessed using experiments. However, in cases when experimental manipulations are logistically or ethically unfeasible, SEM can be fitted on correlative survey data, and when combined with prior knowledge on causal interactions, used to identify the direct and/or indirect paths that are most likely to govern the system. In cases when the direction of causality is uncertain or can change over time/space, SEM fitted on longitudinal data (multiple measures over time) increases the ability to identify the most plausible direction of causality [29].

The aim of this study was to explore the relationships between aquatic vegetation and turbidity and how these relationships vary with season and spatial scale, using field survey data combined with causal modeling. We conducted a large-scale field survey in 32 shallow bays along the central Swedish Baltic Sea coast, which together formed gradients in land-derived nutrient loading and topographic openness of the bays towards the sea. To assess the relationships between vegetation cover and turbidity at both local and regional scale (within and across bays), we sampled aquatic vegetation and turbidity in 6–8 stations per bay. To assess the direct and indirect relationships between land-derived nutrient load, particle load from run-off (salinity as proxy), turbidity and vegetation cover, we used piecewise structural equation modeling [30]. We hypothesized that aquatic vegetation reduces turbidity at both local and regional scale but more so in summer than in spring, due to seasonal variation in vegetation cover [31]. Our results show that the relationships between aquatic vegetation and turbidity differs between seasons and spatial scale of the analyses. We highlight the importance of combining experiments and field surveys encompassing multiple scales and seasons when investigating complex questions at ecosystem level.

## Methods

This study was made in accordance to the ethical regulation laid down in the Swedish ordinance SJVFS 2012:26, which is the Swedish implementation of the Directive 2010/63/EU of the European Parliament and of the Council on the protection of animals used for scientific purposes. The fish sampling procedures applied in the project, described under Survey design and in the Supporting Information, were also judged and approved by the Ethical Board on Animal Experiments of the County Court of Uppsala, Sweden, permit C 139/13. The fish died in the process of lifting the nets.

### Study system

The Baltic Sea is a temperate brackish marginal sea with strong seasonality in solar influx, temperature and biological production. The Baltic Sea is also heavily impacted by anthropogenic activities, most importantly eutrophication from land-based sources (e.g. [32]). A large part of the coastline consists of archipelago areas with shallow, sheltered bays dominated by sediment bottoms. Shallow bays typically harbor a high diversity of aquatic vegetation of both marine and freshwater origin (e.g. [33, 34]). Freshwater species include charophytes (*Chara* spp.) and rooted angiosperms such as pondweeds (*Stuckenia* and *Potamogeton* spp.), water milfoils (*Myriophyllum* spp.), naiads (*Najas marina*) and crowfoots (*Ranunculus* spp.). Marine species include macroalgae such as bladderwrack (*Fucus vesiculosus*), forkweed (*Furcellaria lumbricalis*) and filamentous algae, such as *Cladophora* spp. and *Pylaiella littoralis*.

The Baltic Sea has no tide, and water level fluctuations are mainly caused by air pressure. The topographic openness of shallow bays towards the sea is an important factor that changes over long timescales due to isostatic land uplift [35]. The openness in turn affects key abiotic variables like wave exposure and water retention time, which in turn affects many properties like sedimentation of particles, substrate characteristics, water temperature, and salinity [1, 35].

### Survey design

**Fig 1:**
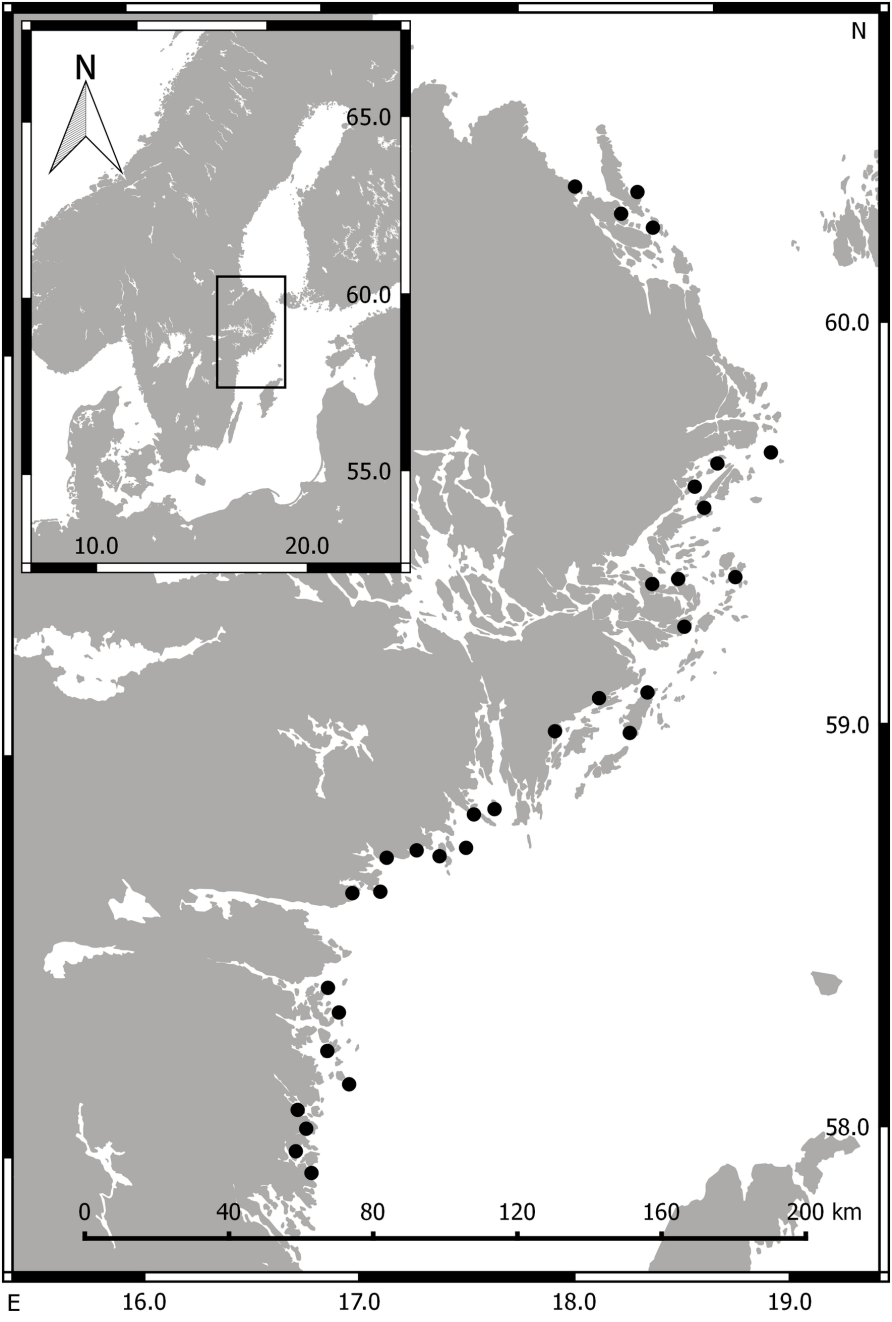
Map of the central Baltic Sea coastline. The position of the 32 shallow bays is marked with black dots. Axis labels show latitude and longitude. The map was created in QGIS v. 2.12.3 [37], with background layers from DeLorme (Esri, Redlands, CA) and the global lakes and wetlands database (level 1) [38].

To explore the relationships between aquatic vegetation and water turbidity, we conducted a field survey in 32 shallow bays situated along a 360 km stretch of the central Swedish Baltic Sea coast (Fig 1). Sampling was done twice; in spring (May), when the annual vegetation has just started to grow and most of the biomass consists of overwintering perennials, and in late summer (August), when the vegetation reaches it maximum cover and biomass [31, 36].

The 32 bays were chosen to together form a gradient in loads of land-derived nutrients (ton year^-1^) from watersheds surrounding each bay, based on fine-scale modelled data (derived from the Swedish Meteorological and Hydrological Institute’s database “SVAR” [version 2012_2] and “S-HYPE” model [version 2012_1_2_1] available at http://www.smhi.se; see [39]). Moreover, the bays were selected to mainly consist of areas shallower than 3 m (to reduce natural variability), and host aquatic vegetation (based on previous surveys of ca 90 percent of the sites). Five of the bays had been surveyed during 4–7 years in the past, and had relatively constant species composition during that period of time.

In each bay, 6–8 stations (higher number with increasing area of the bay) were randomly positioned at 0.5–3 m depth and at least 30 m apart. In total, we sampled 201 stations across the 32 bays. At each station, the percent cover of aquatic vegetation species within a 0.5 × 0.5 m frame was visually estimated by a snorkeler, at three random points in spring and five random points in summer, within a five m radius from each station. The cumulative cover of all submerged vegetation species (excluding filamentous algae) was calculated for each frame (hereafter “vegetation cover”). The measurements were then averaged to give a single value per station and season. Water was sampled at each station at 0.5 m depth for analyses of turbidity, phytoplankton fluorescence, salinity and dissolved nutrients. Water turbidity depends partly on phytoplankton fluorescence, and partly on suspended particles [13]. Since fluorescence is strongly affected by light and temperature, we stored the water samples dark and cold for ca 6 hours until measuring turbidity and fluorescence (three estimates per station), using a handheld turbidi- and fluorometer (Aquafluor®, Turner Designs, USA). Salinity was measured in the field using a Multi 340i voltmeter (WTW, Germany). Water for nutrient analysis was filtered through a 0.45 μm glass-fiber filter, stored dark and cold until freezing, and later analyzed through segmented flow colorimetric analysis [40], with the system ALPKEM FlowSolution IV, OI Analytical, after digestion with acid-persulphate at high temperature (modified after [41]). Our field measurements of dissolved phosphorus and nitrogen indicated that the majority of the bays were nitrogen limited, based on the Redfield ratio [42] (see also [43]). Hence, we chose to include nitrogen (and not phosphorus) in the models. We used the modeled land-derived nitrogen load (ton year^-1^) for two reasons: first, nutrient run-off from land is one of the major causes of eutrophication and high turbidity [44]; second, because it is an exogenous variable (i.e. a variable unaffected by other variables in the model in contrast to e.g. the measured nutrient concentrations which could be influenced by vegetation and phytoplankton), and these typically help identify solvable models. The modelled load of nitrogen and phosphorus were in our data highly correlated (Spearman rho = 0.96, p < 0.0001).

Sediment re-suspension by benthivorous fish has been found to affect turbidity in shallow lakes. The density and activity of benthivorous fish and the sediment type affect resuspension and settling rate of the sediment and further the turbidity [45, 46]. To control for sediment re-suspension by benthivorous fish, four to five Nordic survey gillnets (European Union standardized method EN 14757:2005) were set out in each bay overnight in spring (see detailed description in Supporting Information). There was no correlation between the density of benthivorous fish (black goby, bream, crucian carp, European flounder, ide, roach, rudd and tench, kg/km^2^) and turbidity, but a negative correlation between the density of benthivorous fish and topographic openness (S1 Table).

### Conceptual model

**Fig 2:**
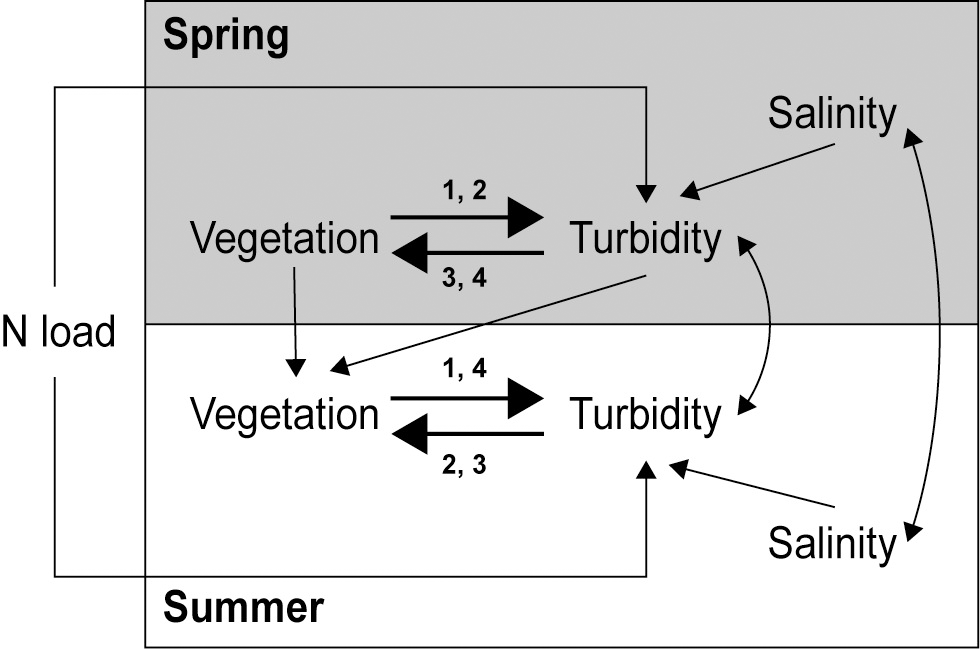
Conceptual model summarizing the four full models (1–4). The models were fitted and compared (using AIC) to assess the strength and direction of the relationships between cover of aquatic vegetation and turbidity in spring (grey box) and summer (white box). The four thicker arrows and respective number combination (1–4) indicate which paths and directions that were included in which model. Double-headed arrows are correlated errors.

Many field surveys suggest that high turbidity reduces plant cover (e.g. by reducing light penetration), but on the other hand, many experiments show that high plant cover can reduce turbidity (e.g. by increasing sediment settlement and/or reducing sediment resuspension). To assess which of these relationships that best fitted our field survey data, and whether these relationships depended on season, we specified (and then evaluated, see below) four alternative conceptual models (model 1–4), each including one of the four possible combinations of directed relationships between vegetation and turbidity in spring vs. summer (Fig 2). For example, model 1 included an effect of vegetation on turbidity in both spring and summer, whereas model 4 included the reverse relationships. Based on prior system knowledge, turbidity was hypothesized to also increase with i) land-derived nitrogen load, due to increased growth of phytoplankton [47–49] and ii) low salinity, as a proxy of transfer of suspended inorganic particles with freshwater runoff [11]. Vegetation cover in summer was allowed to depend on vegetation cover in spring. Correlated errors between spring and summer salinity as well as turbidity were incorporated to account for potential temporal autocorrelation. The topographic openness of bays was included in initial model exploration, since several previous studies have shown that it is one of the most important factors structuring shallow bay biota, including plant assemblages [1, 35, 50]. However, since topographic openness did not affect turbidity, and because our relatively small bay-level (regional scale) sample size (N = 32) restricted the number of parameters that could be included, we excluded topographic openness from the final models.

### Statistical analyses

All statistical analyses were made in R v. 3.2.5 [51]. To achieve normally distributed data, the variables nitrogen load, turbidity and fluorescence were log_10_-transformed, while vegetation cover was square-root transformed. All predictor variables were tested for multicollinearity by i) calculating the variance inflation factor (less than 2) for each predictor [52], and the correlation coefficient (less than 0.6) for all predictor combinations.

To quantify the direct and indirect relationships between nitrogen load, salinity, cover of aquatic vegetation and turbidity, we ran path analyses [27, 28] using the *piecewiseSEM* package [30]. Model fit was assessed using the test of directional separation [53], which tests if there are any paths missing in the model, and if the model would improve by including the missing path(s) [54]. The models were simplified by stepwise removal of the non-significant path(s) in order after highest p-value. The relative fit of the four alternative models was assessed using Akaike’s Information Criterion (AIC). Models with less than 4 units of difference in AIC were assumed to fit the data equally well [55]. To be able to compare the relative strength of the significant paths, standardized path coefficients (scaled by mean and variance) were calculated for the final models [56]. Marginal and conditional R^2^ were calculated for the local scale models (where “bay” was a random factor) (see [57]). Marginal R^2^ explains proportion of variance explained by the fixed factors alone while conditional R^2^ explains the proportion of variance explained by the fixed and random factors.

To assess whether the relationships changed with spatial scale, data were analyzed at two spatial scales; regional (using means per bay, N = 32) and local (using station-scale values [N = 201] including “bay” as random factor with 32 levels). To elucidate through which mechanisms vegetation might affect the turbidity, we then replaced turbidity with i) fluorescence (a proxy for phytoplankton abundance) and ii) sediment-driven turbidity (see below) respectively, and evaluated these models in the same way. Sediment-driven turbidity was estimated by first modeling the effect of fluorescence on turbidity using a simple linear regression (spring regional scale R^2^ = 0.32, p < 0.001, summer regional scale R^2^ = 0.21, p < 0.01). Thereafter, we extracted the residuals from the model (i.e. the variation in turbidity not explained by fluorescence), and used these as an estimate of sediment-driven turbidity.

We acknowledge that we have not measured the actual sediment-driven turbidity, but estimated it as the turbidity that is not explained by fluorescence (i.e. the residuals). The concentration of suspended particulate matter (SPM) can be estimated from turbidity measurements as ln(SPM) = 0.97*Turbidity– 0.081 [58]. However, in our case such approximation would be of little use as SPM is a linear transformation of turbidity values, and would therefore give the same results in our SEM models as when including turbidity.

To test if there were seasonal differences in the variables (excluding nutrient load, which was based on yearly model estimates), we ran a mixed model for each variable with “season” as fixed factor, and “bay” as random factor. Each model was tested against a simplified model without the fixed factor “season”, using the anova function in R. We used the p-value from the likelihood ratio test to assess if the models differed significantly (p < 0.05), in which case "season" was regarded as significant (shown in S2 Table).

## Results

### Relationships at the regional (between-bay) scale

#### Turbidity

**Fig 3:**
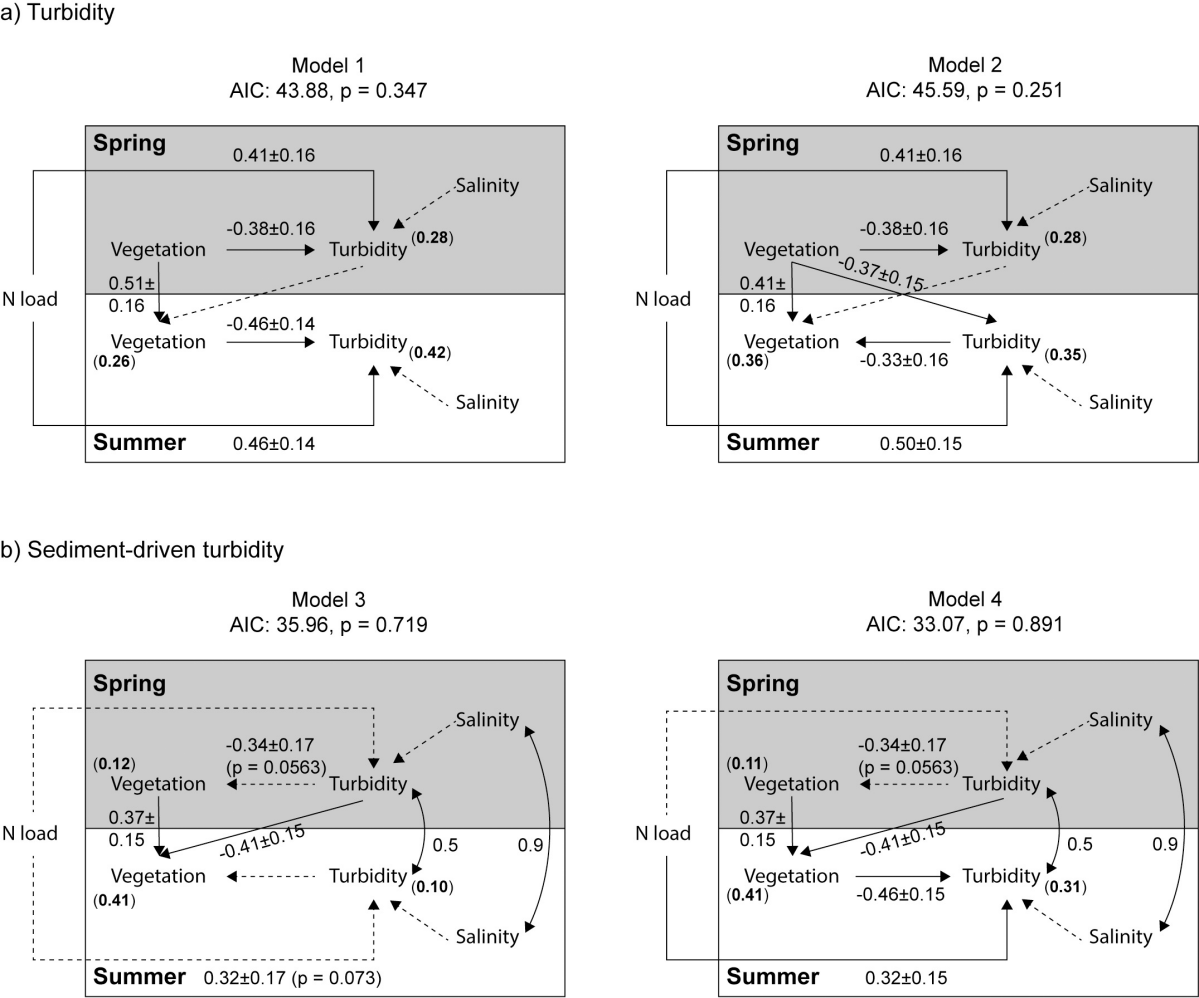
**Path diagrams of the best-fitting regional scale models using a) turbidity and b) sediment-driven turbidity.** Variables within the grey vs. white box were measured in spring vs. summer, respectively. Solid lines indicate significant paths (p < 0.05), while dashed lines indicate non-significant paths. Double-headed arrows show correlated errors. Standardized regression coefficients ± SE are shown for paths with p < 0.1. Bold numbers within brackets show R^2^.

**Fig 4:**
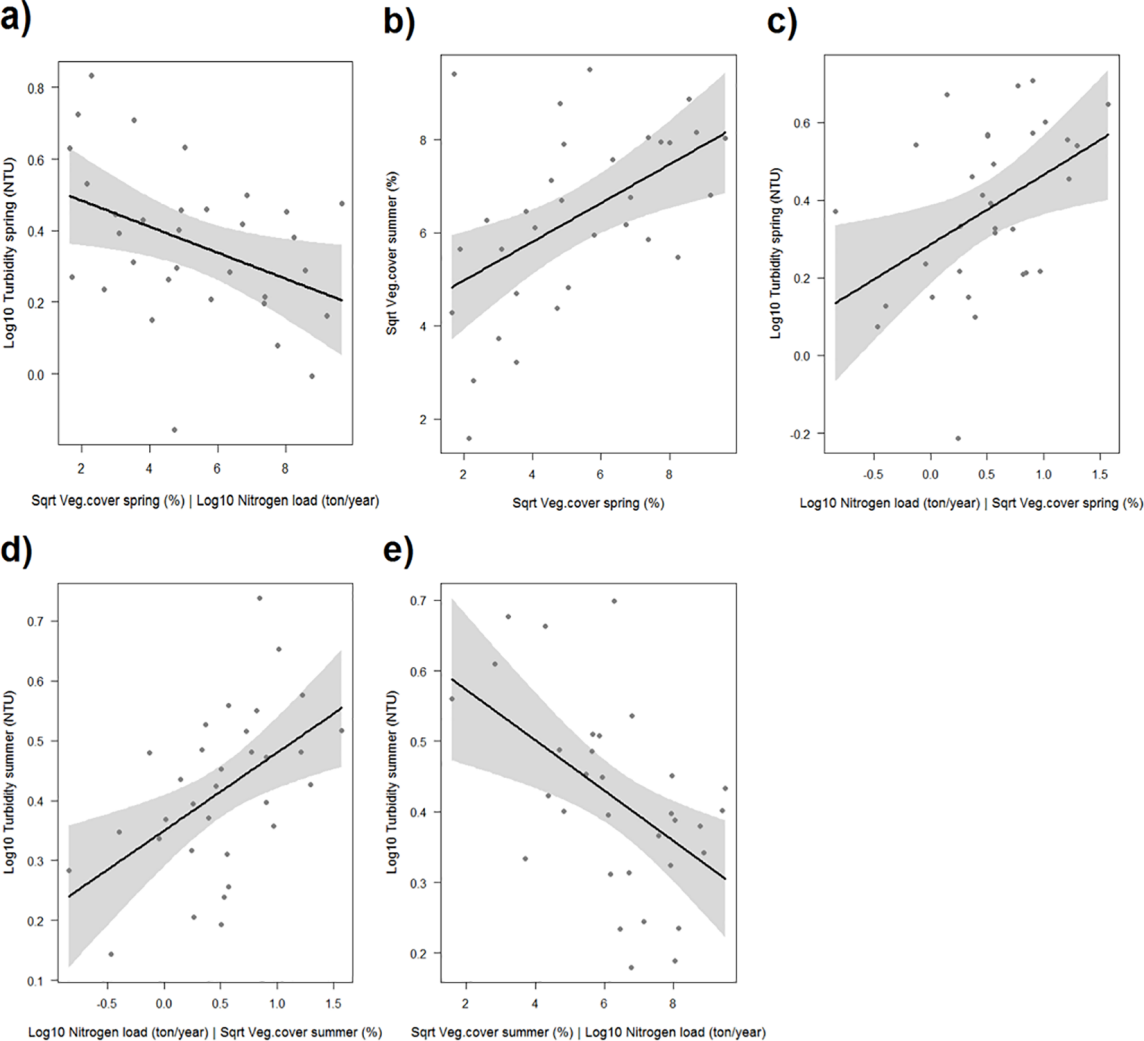
Partial regression plots of the significant relationships from the regional scale analysis of turbidity. The relationships shown are from one of the two best fitting models (model 1). Turbidity and nitrogen load are log_10_-transformed, and vegetation cover is square-root transformed. (a) partial effect of vegetation cover in spring on turbidity in spring, given the effect of the co-variable nitrogen load; (b) effect of vegetation cover in spring on vegetation cover in summer; (c) partial effect of nitrogen load on turbidity in spring, given the effect of the co-variable vegetation cover in spring; (d) partial effect of nitrogen load on turbidity in summer, given the effect of the co-variable vegetation cover in summer; (e) partial effect of vegetation cover in summer on turbidity in summer, given the effect of the co-variable nitrogen load.

**Fig 5:**
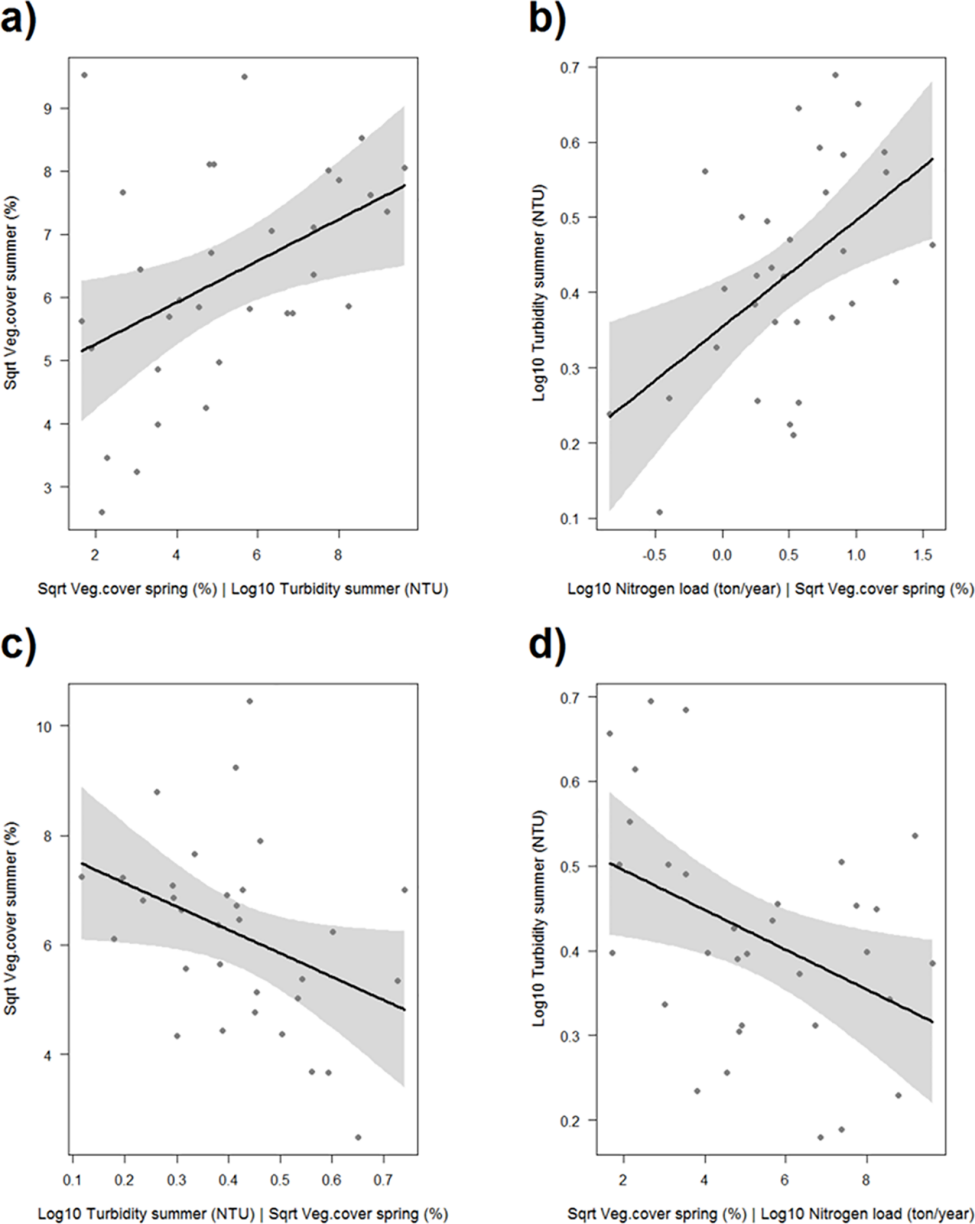
Partial regression plots of the significant relationships from the regional scale analysis of turbidity. The relationships shown are from one of the two best fitting models (model 2). Turbidity and nitrogen load are log_10_-transformed, and vegetation cover is square-root transformed. (a) partial effect of vegetation cover in spring vegetation cover in summer, given the effect of the co-variable turbidity in summer; (b) partial effect of nitrogen load on turbidity in summer, given the effect of the co-variable vegetation cover in spring; (c) partial effect turbidity in summer on vegetation cover in summer, given the effect of the co-variable vegetation cover in spring; (d) partial effect of vegetation cover in spring on turbidity in summer, given the effect of the co-variable nitrogen load. Fig 4A and 4C were also significant in model 2.

Based on the test of d-separation and AIC value, two of the four models fitted the data best; model 1 (AIC: 43.88) and model 2 (AIC: 45.59) (Fig 3A, S3 Table). The two models shared four direct effects; a negative effect of vegetation cover on turbidity in spring (p = 0.023, Fig 4A), a positive effect of spring vegetation on summer vegetation (model 1 p = 0.003, Fig 4B; model 2 p = 0.015, Fig 5A), and positive effects of nitrogen load on turbidity in spring and summer (p = 0.003–0.014, Figs 4C, 4D and 5B). Consequently, the influence of aquatic vegetation on turbidity in spring appeared to be stronger than the effect of turbidity on vegetation. Model 1 also contained a direct negative effect of vegetation on turbidity in summer (p = 0.003, Fig 4E). Model 2 instead included the reverse relationship–a direct negative effect of summer turbidity on summer vegetation (p = 0.042, Fig 5C)–but also a direct negative effect of spring vegetation on summer turbidity (p = 0.020, Fig 5D); a path not included in the initial model, but identified by the test of d-separation as a missing path needed to reach a good fit of the data. When combined, these two paths result in an *indirect* positive effect of spring vegetation on summer vegetation, mediated by summer turbidity. In other words, model 2 suggests high vegetation cover in spring fed back positively on itself over time, by reducing turbidity.

#### Fluorescence

When turbidity was replaced by fluorescence, the four models were identical after model simplification (AIC: 31.08; S3 Table). The only significant relationship was the link from spring vegetation to summer vegetation (p = 0.003, Fig 4B). There was also a trend to a positive effect of nitrogen load on spring and summer fluorescence (p = 0.070–0.072).

#### Sediment-driven turbidity

When turbidity was replaced by sediment-driven turbidity, two of the four models fitted the data best; model 3 (AIC: 35.96) and model 4 (AIC: 33.07) (Fig 3B, S3 Table). The two models shared two direct effects; a negative effect of sediment-driven turbidity in spring on vegetation in summer (p = 0.011, Panel A in S1 Fig), and a positive effect of spring vegetation on summer vegetation (p = 0.021, Panel B in S1 Fig). The two best fitting models also shared two trends; a trend to a negative effect of sediment-driven turbidity in spring on vegetation cover in spring (p = 0.056, Panel C in S1 Fig), and a positive effect (or trend) of nitrogen load on sediment-driven turbidity in summer (model 3: p = 0.073, model 4: p = 0.048, Panel D and E in S1 Fig). Model 4 also contained a direct negative effect of summer vegetation on summer sediment-driven turbidity (p = 0.0061, Panel F in S1 Fig). When combined with the other significant paths (see above), the model indicates there was a positive *indirect* effect of sediment-driven turbidity in spring on sediment-driven turbidity in summer, mediated by vegetation cover in spring and summer. In other words, high sediment-driven turbidity in spring fed back positively on itself over time, by reducing vegetation.

### Relationships at the local (within-bay) scale

#### Turbidity

**Fig 6:**
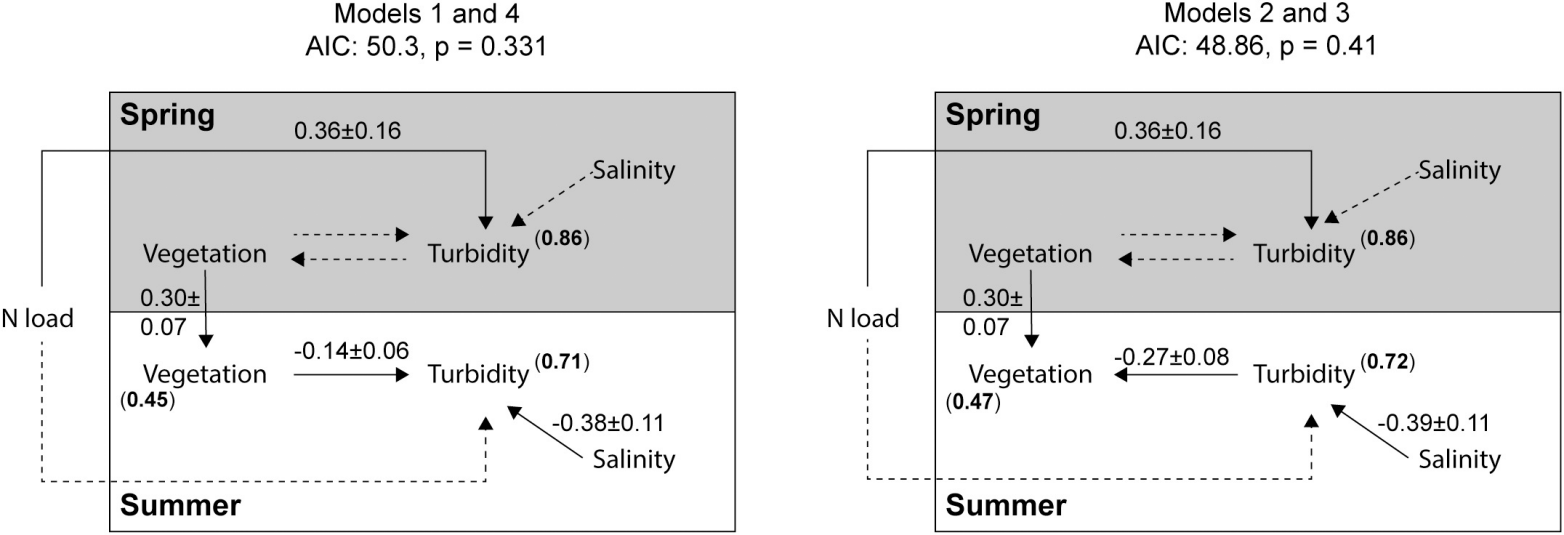
Path diagrams of the four best-fitting models using local-scale (station) data on turbidity. The path diagram to the left shows model 1 and 4, which became identical after model simplification. The path diagram to the right shows model 2 and 3, which also became identical after model simplification. Variables within the grey vs. white box were measured in spring vs. summer, respectively. Solid lines indicate significant paths (p < 0.05) with standardized regression coefficients ± SE, while dashed lines indicate non-significant paths. Bold numbers within brackets show conditional R^2^.

After model selection and simplification model 1 and 4 were identical, and so were model 2 and 3. The two final models fitted the data equally well, i.e. AIC differed with less than 4 units; model 1 and 4 (AIC: 50.30) and model 2 and 3 (AIC: 48.86) (Fig 6; S3 Table). Both models identified a negative relationship between vegetation and turbidity, but with different directions of causality. Both models shared three direct effects; a positive effect of nitrogen load on spring turbidity (p = 0.034), a positive effect of vegetation in spring on vegetation in summer (p < 0.001), and a negative effect of summer salinity on turbidity in summer (p = 0.001). Model 1 and 4 also contained a weak direct negative effect of vegetation on turbidity in summer (p = 0.016), while model 2 and 3 instead included the reverse relationship; a direct negative effect of summer turbidity on summer vegetation (p = 0.001).

#### Fluorescence

When turbidity was replaced by fluorescence, the only significant relationship was a positive effect of spring vegetation on summer vegetation (p < 0.001). However, there was a tendency to a positive effect of nitrogen load on spring and summer fluorescence (p = 0.076). Hence, the four models were identical after model selection (AIC: 37.67; S3 Table).

#### Sediment-driven turbidity

When turbidity was replaced by sediment-driven turbidity, model 1 and 4 were identical after model selection and simplification, and so were model 2 and 3. The two final models fitted the data equally well, i.e. AIC differed with less than 4 units; model 1 and 4 (AIC: 16.55) and model 2 and 3 (AIC: 12.70) (S3 Table). The two final models contained a positive effect of spring vegetation on summer vegetation (p < 0.0001). The only other significant path in the models was a negative effect of sediment-driven turbidity in summer on summer vegetation (p = 0.0026, model 2 and 3), i.e. the opposite relationship than at the regional scale.

### Seasonal differences in endogenous variables

Vegetation cover, salinity and fluorescence were higher in summer than in spring, at both local and regional scales (S2 Table). Meanwhile, there was no seasonal difference in turbidity at the regional scale or sediment-driven turbidity at either regional or local scale. Turbidity was slightly higher in summer than in spring, at local scale. The variable with the highest seasonal difference was vegetation cover (estimate of the fixed effect season 1.06 at regional scale and 1.22 at local scale, S2 Table). The ranges of the variables land-derived nitrogen and phosphorus load, dissolved nutrients (NH_4_, PO_4_ and sum of NO_2_ and NO_3_), salinity, turbidity, fluorescence and vegetation cover in spring and summer at regional scale (averages/bay) are shown in Table 1.

**Table 1:** Ranges of the measured and modelled variables at regional scale (averages/bay) in spring and summer respectively.

Variable	Description	Season	Range
Load.N_ton/year	Modelled net nitrogen load (ton/year) exported to the sea from the watersheds surrounding the bay*	one yearly value	0.1435–37.2628
Load.P_ton/year	Modelled net phosphorus load (ton/year) exported to the sea from the watersheds surrounding the bay*	one yearly value	0.0036–1.5483
NH_4_	NH4-N μmol/l	spring	0.23–2.84
		summer	0.22–2.41
PO_4_	PO4-P μmol/l	spring	0.04–0.30
		summer	0.02–0.69
NO_2_ and NO_3_	(NO_2_+NO_3_)-N μmol/l	spring	0.06–0.86
		summer	0.05–0.75
Salinity	Measured salinity (PSU)	spring	3.3–6.8
		summer	4.8–6.7
Turbidity	Measured turbidity value (NTU)	spring	0.6–6.4
		summer	1.3–5.5
Fluorescence	Measured fluorescence value (RFU)	spring	80.3–327.5
		summer	175.4–337.0
Vegetation cover	Percentage of coverage of all submerged aquatic vegetation species excluding filamentous algae	spring	3–93
		summer	3–89

*derived from the Swedish Meteorological and Hydrological Institute’s database “SVAR” [version 2012_2] and “S-HYPE” model [version 2012_1_2_1] available at http://www.smhi.se; see [39].

The most common vegetation species (occurring in most bays) in spring and summer was *Stuckenia pectinata*, followed by *Fucus vesiculosus* and *Chorda filum* in spring and *Potamogeton perfoliatus* and *Fucus vesiculosus* in summer (Table 2).

**Table 2:** Vegetation species recorded in spring and summer. Average cover of each species over all 201 stations and standard deviation (SD) for spring and summer. Species are ordered after highest average cover over all stations in summer. Numbers below 1 are rounded to the closest decimal. The rightmost column shows the number of bays (out of the 32) that the species were recorded in, spring values are shown in brackets.

Species	Spring		Summer		Recorded in no. of bays
	Average cover (%)	SD	Average cover (%)	SD	
*Fucus vesiculosus*	18	± 31	15	± 26	22 (23)
*Stuckenia pectinata*	4	± 11	8	± 14	29 (25)
*Najas marina*	0	± 0	6	± 22	15 (0)
*Potamogeton perfoliatus*	1	± 3	3	± 8	24 (17)
*Myriophyllum spicatum*	0.6	± 3	2	± 6	22 (8)
*Ruppia cirrhosa*	0.8	± 3	2	± 6	11 (7)
*Ceratophyllum demersum*	0.3	± 3	1	± 6	14 (9)
*Vaucheria dichotoma*	0.7	± 2	1	± 11	2 (2)
*Monostroma balticum*	3	± 8	1	± 8	2 (4)
*Chara baltica*	0.2	± 2	1	± 6	13 (5)
*Ranunculus circinatus*	0.7	± 3	1	± 5	14 (11)
*Myriophyllum sibiricum*	0.3	± 1	0.9	± 4	11 (8)
*Zannichellia palustris*	0.8	± 4	0.5	± 2	14 (13)
*Callitriche hermaphroditica*	0.2	± 1	0.5	± 2	12 (7)
*Chara tomentosa*	0.3	± 2	0.3	± 2	3 (2)
*Chorda filum*	3	± 9	0.2	± 1	8 (20)
*Furcellaria lumbricalis*	0.4	± 4	0.2	± 2	3 (3)
*Chara aspera*	0.06	± 0.8	0.2	± 1	3 (2)
*Chara globularis*	0.002	± 0.02	0.05	± 0.6	3 (1)
*Chara canescens*	0	± 0	0.02	± 0.3	1 (0)
*Chara horrida*	0	± 0	0.007	± 0.1	1 (0)
*Hippuris vulgaris*	0.1	± 1	0.006	± 0.07	1 (2)
*Lemna trisulca*	0	± 0	0.005	± 0.07	1 (0)
*Potamogeton pusillus*	0	± 0	0.005	± 0.07	1 (0)
*Ranunculus peltatus* ssp. *baudotii*	0.09	± 1	0.004	± 0.05	2 (3)
*Monostroma grevillei*	0.007	± 0.09	0	± 0	0 (1)
*Ruppia maritima*	0.02	± 0.2	0	± 0	0 (2)
*Tolypella nidifica*	0.06	± 0.3	0	± 0	0 (6)

## Discussion

The aim of this study was to explore the direction and strength of the relationships between aquatic vegetation and water turbidity, and how these relationships vary with season (spring vs. summer) and spatial scale (regional vs. local). Based on field survey data collected along a 360 km coastline in the Baltic Sea, we found that structural equation models including a negative effect of spring vegetation cover on spring turbidity fitted the data better than models including the reverse relationship. Moreover, the best-fitting models suggested that summer vegetation either regulated summer turbidity, or that high spring vegetation cover, by upholding low turbidity during the season, could facilitate high summer vegetation cover; i.e. a positive feedback effect by vegetation on itself. The effect of vegetation on turbidity (-0.46) was comparable in strength to the effect of nitrogen load on turbidity (0.41) at regional scale. In models instead exploring the relationships between vegetation and sediment-driven turbidity, one of the best-fitting models at regional scale included a negative effect of sediment-driven turbidity in spring on vegetation cover in summer, which in turn had a negative effect on sediment-driven turbidity in summer, indicating a positive feedback of sediment-driven turbidity on itself. This suggests that high sediment-driven turbidity in spring decreased the growth of vegetation during the season, and thereby the regulating effect of summer vegetation on sediment-driven turbidity in summer. Another potential mechanism is that wind-induced turbidity in spring leads to burial of propagules and thereby a lower vegetation cover in summer [59]. However, in the present study, vegetation cover was higher in more exposed, than sheltered, bays in spring (S4 Table)–hence, burial of propagules at exposed sites is not likely to affect vegetation cover in our study. We acknowledge that the relationship between wind exposure and turbidity might be more complex with an interacting effect of wave exposure, depth and substrate characteristics on vegetation cover, i.e. that wind exposure has a stronger effect on turbidity in shallow areas with finer sediment [13]. Yet, in our coastal ecosystem, the propagules are more likely to be affected by ice scoring and freshwater outflow that brings suspended particles (we used salinity as proxy in the models) than by wind-induced turbidity [11, 60].

Based on regional-scale data, the models including a negative effect of high vegetation cover on turbidity in spring fitted the data much better than models including the reverse relationship (AIC differed with more than 4 units). Consequently, the effect of aquatic vegetation on turbidity appears to be stronger than the reverse effect; a finding that contrasts with results found in many previous field surveys, where high turbidity has been argued to regulate aquatic vegetation (e.g. [7–10]). Our study is, however, not alone in suggesting a strong effect of aquatic vegetation on water quality. Several large-scale surveys and field experiments have shown that the marine plant eelgrass (*Zostera marina* L.) can be a considerable nutrient sink and stabilize sediments, and thereby improve water quality over large scales [18, 61, 62]. One potential explanation for the contrasting conclusions about the relationship between vegetation and turbidity in the past could be that many studies reporting a negative effect of turbidity on aquatic vegetation have used simple, bivariate statistical analyses that explores net relationships, and have not specifically assessed the direction of causality, or the strength of indirect (mediated) relationships. There are, however, correlative field studies and experiments where causation is clear; for example, turbidity has been found to limit the depth distribution of aquatic vegetation in lakes [63], the cover of aquatic vegetation has decreased over time, following increased turbidity due to eutrophication [9], and successful restoration of seagrass meadows has strongly reduced local turbidity [62]. Meanwhile, many of the studies that do explore the direction of causality find that also in field data, there appears to be a substantial effect of aquatic vegetation on turbidity, at least during periods of high growth. Therefore, we–as others before [24, 26]–encourage the use of targeted experiments and/or system-level analyses (including structural equation modeling) when addressing complex questions at ecosystem level, such as the dual relationship between aquatic vegetation and turbidity.

One of the best-fitting regional-scale models also contained an indirect positive effect of vegetation cover in spring on vegetation cover in summer; a positive (self-enhancing) feedback mechanism similar to those recently reviewed by Maxwell et al. [64] and Adams et al. [65]. Positive feedback mechanisms can play a pivotal role for seagrass ecosystem dynamics [64], and our results indicate that also submerged aquatic vegetation in the brackish Baltic Sea may positively affect its own conditions [50]. Meanwhile, the best-fitting models that instead included sediment-driven turbidity suggested that sediment-driven turbidity in spring either regulated summer vegetation, or that high sediment-driven turbidity in spring–by reducing vegetation in summer–increased sediment-driven turbidity in summer; i.e. a positive feedback of sediment-driven turbidity on itself. These two opposing yet complementary feedback mechanisms points to the possible existence of two alternative regimes or states in shallow coastal areas of the Baltic Sea; one in which high cover of vegetation is self-sustaining by reducing total turbidity, and one in which sediment-driven turbidity is self-sustaining by reducing vegetation cover. Such alternative regimes have previously been described in shallow lakes [66] and the Dutch Wadden Sea [67], and have also been proposed for shallow bays in the Baltic Sea [68]. Positive feedbacks are necessary for alternative regimes to be self-sustaining, but do not confirm their existence (for a recent review see [64]). Consequently, our results should be seen as an interesting indication, but further studies are needed to explore whether positive feedbacks can trigger shifts between alternative regimes in shallow coastal areas of the Baltic Sea. Such studies should in our view focus on identifying i) the exact mechanisms involved, ii) potential thresholds in vegetation cover and nutrient load, iii) if and how such threshold levels vary in space and over time, and iv) which vegetation traits that are most important for regulating turbidity.

In contrast to the models including turbidity and sediment-driven turbidity, we found no relation between fluorescence and vegetation. One reason could be that fluorescence is highly influenced by small-scale and short-term variability in light and temperature [69, 70]. Water temperature in our study system is higher during spring and summer in enclosed bays with high retention time [1], whereas spring vegetation cover is lower. In this study, retention time and/or bay openness could not be added to the regional scale models, due to the limited sample size (N = 32) and relatively complex models. However, studies from north European lakes suggest that phytoplankton do not contribute to more than 50 percent of the turbidity [66], and could be a reason for failing bio-manipulation in shallow lakes via top-down control [71]. The lack of strong relationships including fluorescence highlights the need to identify other factors that can influence turbidity and vegetation and their relation, using e.g. experiments.

Turbidity is not only influenced by phytoplankton fluorescence and suspended particulate matter (SPM), but also by colored dissolved organic matter (CDOM) or humic substances [72]. The brackish Baltic Sea has a relatively high CDOM concentration in relation to marine waters, but the concentration varies considerably within the Baltic Sea. For example, in our study area (coastal northern Baltic Proper) CDOM is lower and less variable than in the coastal Gulf of Bothnia [73]. CDOM was not measured in this study, and should be included in future studies to better assess the relative contribution of different sources to turbidity and plant-turbidity relationships. But since we measured turbidity at 530 nm, and CDOM mainly absorbs light at shorter wavelengths (the ultraviolet and blue regions of the electromagnetic spectrum), and the absorption declines exponentially with increasing wavelength [74], our turbidity measurements are not likely to be influenced by CDOM. Moreover, while CDOM can be an indicator of fresh water inflow or decay of organic matter in the littoral zone [75], we instead used salinity as a proxy for freshwater runoff. Salinity appeared to negatively influence turbidity at the local (within-bay) scale, but not at the regional (between-bay) scale. Together, these results indicate that while freshwater runoff (most likely including CDOM) can affect turbidity locally (for example near freshwater outlets), it is not a major contributor to turbidity across the studied bays.

The relatively clear relationships found in the regional scale analyses did not appear when we instead used local scale data (i.e. within bays). This difference could in theory be explained by several, not mutually exclusive mechanisms. First, environmental conditions are spatially heterogeneous even at small scale [76], which might be reflected by the high unexplained variability in our variables at local scale. In our system, the cover of aquatic vegetation is heterogeneous at small (within-bay) scales, and much of this heterogeneity is reduced in the regional scale analyses, since means per bay were used. Second, even though the influence of organisms on the surrounding environment is often spatially limited [76], the spatial scale at which an organism affects its environment varies strongly between species. We have, for example, recently found that the indirect positive effect of macroinvertebrate grazers on vegetation, through their grazing of ephemeral filamentous macroalgae, is limited to the local (within-bay) scale, while the indirect positive effect of large predatory fish on macroinvertebrate grazers, through their predation of mesopredatory fish, occurs at the regional (between-bay) spatial scale. This cross-scale interaction could entangle mechanisms for the observed decrease of ephemeral algae with increasing biomass of large predatory fish, and the positive relationship between mesopredatory fish and ephemeral algae, at the larger scale [77]. Consequently, it is possible that the relative importance of organisms also at larger (e.g. regional) scale has been underestimated in previous studies.

In summary, our results suggest that in spring, and possibly in summer, high cover of aquatic vegetation plays an important role by reducing water turbidity. Consequently, the mechanisms by which aquatic plants are known to regulate turbidity in small scale experimental studies [3, 4, 16, 17], may also hold across large spatial scale (see also [18]). From a coastal management perspective, this indicates that in addition to reducing nutrient loads into coastal areas, the protection and (if needed) restoration of submerged aquatic vegetation could help maintain high water quality in shallow coastal areas.

## Supporting information

S1 Text Description of the gillnet fishing in spring and how the relationship between turbidity and benthivorous fish was tested. (PDF)

S1 Table Average CPUE over all bays of the fish species caught in gillnets in spring. The species are sorted after the highest average CPUE. Benthivorous fish species are marked in bold.
(PDF)

S2 Table Results from the analyses of seasonal differences in variables. The table shows the likelihood ratio test statistic (LRT), its p-value, the estimate and standard error of the fixed effect “Season” (level: summer) of the mixed models, for all variables at regional scale (n = 32) and local scale (N = 201).
(PDF)

S3 Table Results from the directional separation test for model 1–4 at local and regional scale. At regional scale averages/bay were used. Bold numbers indicate the best fitting simplified models. Models that differ with less than 4 units of AIC are considered to fit the data equally well.
(PDF)

S4 Table Correlation matrix for the regional scale data, with means per bay (n = 32). Pearson correlation coefficients are shown below the diagonal, p-values are shown above the diagonal. Bold numbers show significant correlations. Topographic openness, retention time, nitrogen load, fluorescence and turbidity are log10-transformed, vegetation cover is square-root transformed.
(PDF)

S1 Fig Partial regression plots of the significant relationships and tendencies from the regional scale analysis of sediment-driven turbidity. The sediment-driven turbidity is the residuals of turbidity predicted by fluorescence. The relationships shown are from the two best fitting models (model 3 and 4). Nitrogen load is log_10_-transformed, and vegetation cover is square-root transformed. (a)partial effect of sediment-driven turbidity in spring on vegetation cover in summer, given the effect of the co-variable vegetation cover in spring; (b) partial effect of vegetation cover in spring on vegetation cover in summer, given the effect of the co-variable sediment-driven turbidity in spring; (c) effect of sediment-driven turbidity in spring on vegetation cover in spring (p = 0.0563); (d) effect of nitrogen load on sediment-driven turbidity in summer in model 3 (p = 0.0730); (e) partial effect of nitrogen load on sediment-driven turbidity in summer, given the effect of the co-variable vegetation cover in summer in model 4; (f) partial effect of vegetation cover in summer on sediment-driven turbidity in summer, given the effect of the co-variable nitrogen load in model 4.
(TIF)

S1 Appendix Original field data, including metadata. Field data of vegetation cover, turbidity, fluorescence, salinity and dissolved nutrients at station level (N = 201) and bay level data (n = 32) of topographic openness, retention time and latitude. For more detailed descriptions of the variables see the metadata sheet in S1 Appendix.
(XLSX)

## References

[1] Snickars M, Sandström A, Lappalainen A, Mattila J, Rosqvist K, Urho L. Fish assemblages in coastal lagoons in land-uplift succession: The relative importance of local and regional environmental gradients. Estuar Coast Shelf Sci. 2009;81(2):247–56.

[2] Snickars M, Sundblad G, Sandström A, Ljunggren L, Bergström U, Johansson G, et al Habitat selectivity of substrate-spawning fish: Modelling requirements for the Eurasian perch Perca fluviatilis. Mar Ecol Prog Ser. 2010;398:235–43.

[3] Benoy GA, Kalff J. Sediment accumulation and Pb burdens in submerged macrophyte beds. Limnol Oceanogr. 1999;44(4):1081–90.

[4] Kufel L, Ozimek T. Can Chara control phosphorus cycling in Lake Luknajno (Poland)? Hydrobiologia. 1994;275/276(1991):277–83.

[5] Iverson RL, Bittaker HF. Seagrass distribution and abundance in Eastern Gulf of Mexico coastal waters. Estuar Coast Shelf Sci. 1986;22(5):577–602.

[6] Dennison WC. Effects of light on seagrass photosynthesis, growth and depth distribution. Aquat Bot. 1987;27(1):15–26.

[7] Duarte CM. Seagrass depth limits. Aquat Bot. 1991;40(4):363–77.

[8] Sand-Jensen K, Lagergaard Pedersen N, Thorsgaard I, Moeslund B, Borum J, Brodersen KP. 100 years of vegetation decline and recovery in Lake Fure, Denmark. J Ecol. 2008;96(4):260–71.

[9] Krause-Jensen D, Carstensen J, Dahl K, Bäck S, Neuvonen S. Testing relationships between macroalgal cover and Secchi depth in the Baltic Sea. Ecol Indic. 2009;9(6):1284–7.

[10] Krause-Jensen D, Carstensen J, Nielsen SL, Dalsgaard T, Christensen PB, Fossing H, et al Sea bottom characteristics affect depth limits of eelgrass Zostera marina. Mar Ecol Prog Ser. 2011;425:91–102.

[11] Olesen B. Regulation of light attenuation and eelgrass Zostera marina depth distribution in a Danish embayment. Mar Ecol Prog Ser. 1996;134:187–94.

[12] Carpenter SR, Caraco NF, Correll DL, Howarth RW, Sharpley AN, Smith VH. Nonpoint pollution of surface waters with phosphorus and nitrogen. Ecol Appl. 1998;8(3):559–68.

[13] Scheffer M. Ecology of Shallow Lakes. DeAngelis DL, Manly BFJ, editors. (First edition 1998): Springer; 2004. 378 p.

[14] Lou J, Ridd P V. Modelling of suspended sediment transport in coastal areas under waves and currents. Estuar Coast Shelf Sci. 1997;45:1–16.

[15] Hilton BYJ, Phillips GL. The effect of boat activity on turbidity in a shallow broadland river. J Appl Ecol. 1982;19:143–50.

[16] Nepf HM, Koch EW. Vertical secondary flows in submersed plant-like arrays. Limnol Oceanogr. 1999;44(4):1072–80.

[17] Wium-Andersen S, Anthoni U, Christophersen C, Houen G. Allelopathic effects on phytoplankton by substances isolated from aquatic macrophytes (Charales). Oikos. 1982;39:187–90.

[18] van der Heide T, van Nes EH, van Katwijk MM, Olff H, Smolders AJP. Positive feedbacks in seagrass ecosystems—evidence from large-scale empirical data. PLoS One. 2011 1;6(1):e16504 doi: 10.1371/journal.pone.0016504 21283684 10.1371/journal.pone.0016504 PMC3025983

[19] Koch EW, Barbier EB, Silliman BR, Reed DJ, Perillo GME, Hacker SD, et al Non-linearity in ecosystem services: temporal and spatial variability in coastal protection. Front Ecol Environ. 2009;7(1):29–37.

[20] Chen S, Sanfod LP, Koch EW, Shi F, North EW. A nearshore model to investigate the effects of seagrass bed geometry on wave attenuation and suspended sediment transport. Estuaries and Coasts. 2007;30(2):296–310.

[21] Blindow I, Hargeby A, Andersson G. Seasonal changes of mechanisms maintaining clear water in a shallow lake with abundant Chara vegetation. Aquat Bot. 2002;72:315–34.

[22] Tokoro T, Hosokawa S, Miyoshi E, Tada K, Watanabe K, Montani S, et al Net uptake of atmospheric CO2 by coastal submerged aquatic vegetation. Glob Chang Biol. 2014;20(6):1873–84. doi: 10.1111/gcb.12543 24623530 10.1111/gcb.12543 PMC4237463

[23] Gopal B, Sharma KP. Ecology of plant populations In: Gopal B, editor. Ecology and management of aquatic vegetation in the Indian subcontinent. Dordrecht: Kluwer Academic Publishers; 1990 p. 79–106.

[24] Grace JB, Anderson TM, Seabloom EW, Borer ET, Adler PB, Harpole WS, et al Productivity and plant species richness. Nature. 2016;529(7586):390–4. doi: 10.1038/nature16524 26760203 10.1038/nature16524

[25] van de Koppel J, Bouma TJ, Herman PMJ. The influence of local- and landscape-scale processes on spatial self-organization in estuarine ecosystems. J Exp Biol. 2012;215:962–7. doi: 10.1242/jeb.060467 22357589 10.1242/jeb.060467

[26] Alsterberg C, Eklöf JS, Gamfeldt L, Havenhand JN, Sundbäck K. Consumers mediate the effects of experimental ocean acidification and warming on primary producers. Proc Natl Acad Sci U S A. 2013;110(21):8603–8. doi: 10.1073/pnas.1303797110 23630263 10.1073/pnas.1303797110 PMC3666745

[27] Shipley B. Cause and Correlation in Biology: A user’s guide to path analysis, structural equations and causal inference Cambridge, UK: Cambridge University Press; 2000. 63 p.

[28] Grace JB, Anderson TM, Olff H, Scheiner SM. On the specification of structural equation models for ecological systems. Ecol Monogr. 2010;80(1):67–87.

[29] Bullock HE, Harlow LL, Mulaik SA. Causation issues in structural equation modeling research. Struct Equ Model A Multidiscip J. 1994;1(3):253–67.

[30] Lefcheck JS. piecewiseSEM: Piecewise structural equation modeling in R for ecology, evolution, and systematics. Methods Ecol Evol. 2016;7(5):573–9.

[31] Rosqvist K. Distribution and role of macrophytes in coastal lagoons: Implications of critical shifts [Internet]. Åbo Akademi University; 2010 Available from: https://www.doria.fi/bitstream/handle/10024/66626/rosqvist_kajsa.pdf?sequence=1

[32] Gustafsson BG, Schenk F, Blenckner T, Eilola K, Meier HEM, Müller-Karulis B, et al Reconstructing the development of Baltic Sea eutrophication 1850–2006. Ambio. 2012;41(6):534–48. doi: 10.1007/s13280-012-0318-x 22926877 10.1007/s13280-012-0318-x PMC3428479

[33] Munsterhjelm R. The aquatic macrophyte vegetation of flads and gloes, S coast of Finland. Acta Bot Fenn. 1997;157:1–68.

[34] Hansen JP, Snickars M. Applying macrophyte community indicators to assess anthropogenic pressures on shallow soft bottoms. Hydrobiologia. 2014;738(1):171–89.

[35] Hansen JP. Effects of shore-level displacement on the ecology of Baltic Sea bays. Stockholm; 2013.

[36] Berglund J, Mattila J, Rönnberg O, Heikkilä J, Bonsdorff E. Seasonal and inter-annual variation in occurrence and biomass of rooted macrophytes and drift algae in shallow bays. Estuar Coast Shelf Sci. 2003;56:1167–75.

[37] Team QD. QGIS Geographic Information System. Open Source Geospatial Foundation Project [Internet]. 2016 Available from: http://www.qgis.org

[38] Lehner B, Döll P. Development and validation of a global database of lakes, reservoirs and wetlands. J Hydrol. 2004;296(1–4):1–22.

[39] Arheimer B, Dahne J, Donnelly C, Lindström G, Strömqvist J. Water and nutrient simulations using the HYPE model for Sweden vs. the Baltic Sea basin—influence of input-data quality and scale. Hydrol Res. 2012;43(4):315–29.

[40] Koroleff F. Determination of phosphorus In: Grasshoff K. Ehrhardt M. Kremling K, editor. Methods of seawater analysis. 2nd ed. Weinheim: Verlag Chemie; 1983 p. 125–39.

[41] Valderrama JC. The simultaneous analysis of total nitrogen and total phosphorus in natural waters. Mar Chem. 1981;10(2):109–22.

[42] Redfield AC. On the proportions of organic derivations in sea water and their relation to the composition of plankton In: Daniel RJ, editor. James Johnstone Memorial Volume. University Press of Liverpool; 1934 p. 176–92.

[43] Blomqvist S, Gunnars A, Elmgren R. Why the limiting nutrient differs between temperate coastal seas and freshwater lakes: A matter of salt. Limnol Oceanogr. 2004;49(6):2236–41.

[44] Smith VH. Eutrophication of freshwater and coastal marine ecosystems a global problem. Environ Sci Polllution Res. 2003;10(2):126–39. 10.1065/espr2002.12.142 12729046

[45] Meijer ML, Raat AJP, Doef RW. Restoration by biomanipulation of Lake Bleiswijkse Zoom the Netherlands first results. Hydrobiol Bull. 1989;23(1):49–57.

[46] Breukelaar AW, Lammens, Eddy HRR, Klein Breteler JGP, Tátrai I. Effects of benthivorous bream (Abramis brama) and carp (Cyprinus carpio) on sediment resuspension and concentrations of nutrients and chlorophyll a. Freshw Biol. 1994;32(1):113–21.

[47] Jupp BP, Spence DHN. Limitations on macrophytes in a eutrophic lake, Loch Leven. I. Effects on phytoplankton. J Ecol. 1977;65:175–86.

[48] Sand-Jensen K, Borum J. Interactions among phytoplankton, periphyton, and macrophytes in temperate freshwaters and estuaries. Aquat Bot. 1991;41(1–3):137–75.

[49] Baastrup-Spohr L, Lønsmann Iversen L, Dahl-Nielsen J, Sand-Jensen K. Seventy years of changes in the abundance of Danish charophytes. Freshw Biol. 2013;58(8):1682–93.

[50] Rosqvist K, Mattila J, Sandström A, Snickars M, Westerbom M. Regime shifts in vegetation composition of Baltic Sea coastal lagoons. Aquat Bot. 2010;93(1):39–46.

[51] R Core Team. R: A language and environment for statistical computing [Internet]. Vienna, Austria: R Foundation for Statistical Computing; 2016 Available from: https://www.r-project.org/

[52] Montgomery DC, Peck EA. Introduction to linear regression analysis 2nd ed. New York: Wiley; 1992. 527 p.

[53] Shipley B. Confirmatory path analysis in a generalized multilevel context. Ecology. 2009;90(2):363–8. 19323220 10.1890/08-1034.1

[54] Shipley B. The AIC model selection method applied to path analytic models compared using a d-separation test R eports R eports. Ecology. 2013;94(3):560–4. 23687881 10.1890/12-0976.1

[55] Burnham KP, Anderson DR, Huyvaert KP. AIC model selection and multimodel inference in behavioral ecology: Some background, observations, and comparisons. Behav Ecol Sociobiol. 2011;65:23–35.

[56] Grace J. Structural equation modeling and natural systems 1st ed. Cambridge, UK: Cambridge University Press; 2006. 365 p.

[57] Nakagawa S, Schielzeth H. A general and simple method for obtaining R2 from generalized linear mixed-effects models. Methods Ecol Evol. 2013;4:133–42.

[58] Kari E, Kratzer S, Beltrán-Abaunza JM, Harvey ET, Vaičiūtė D. Retrieval of suspended particulate matter from turbidity–model development, validation, and application to MERIS data over the Baltic Sea data over the Baltic Sea. Int J Remote Sens. 2017;38(7):1983–2003.

[59] Cabaço S, Santos R, Duarte CM. The impact of sediment burial and erosion on seagrasses: A review. Estuar Coast Shelf Sci. 2008;79(3):354–66.

[60] Kautsky L. Life strategies of aquatic soft bottom macrophytes. Oikos. 1988;53(1):126–35.

[61] Moore KA. Influence of seagrasses on water quality in shallow regions of the lower Chesapeake Bay. J Coast Res. 2004;45:162–78.

[62] Orth RJ, Moore KA, Marion SR, Wilcox DJ, Parrish DB. Seed addition facilitates eelgrass recovery in a coastal bay system. Mar Ecol Prog Ser. 2012;448:177–95.

[63] Chambers PA, Kalff J. Depth distribution and biomass of submerged macrophyte communities in relation to Secchi depth. Can J Fish Aquat Sci. 1985;42:701–9.

[64] Maxwell PS, Eklöf JS, Katwijk MM Van, O’Brien KR, Torre-Castro M de la, Boström C, et al The fundamental role of ecological feedback mechanisms for the adaptive management of seagrass ecosystems–a review. Biol Rev. 2016;92(3):1521–38. doi: 10.1111/brv.12294 27581168 10.1111/brv.12294

[65] Adams MP, Hovey RK, Hipsey MR, Bruce LC, Ghisalberti M, Lowe RJ, et al Feedback between sediment and light for seagrass: Where is it important? Limnol Oceanogr. 2016;61(6):1937–55.

[66] Scheffer M, Hosper SH, Meijer ML, Moss B, Jeppesen E. Alternative equilibria in shallow lakes. Trends Ecol Evol. 1993;8(8):275–9. doi: 10.1016/0169-5347(93)90254-M 21236168 10.1016/0169-5347(93)90254-M

[67] van der Heide T, van Nes EH, Geerling GW, Smolders AJP, Bouma TJ, van Katwijk MM. Positive feedbacks in seagrass ecosystems: Implications for success in conservation and restoration. Ecosystems. 2007;10:1311–22.

[68] Dahlgren S, Kautsky L. Can different vegetative states in shallow coastal bays of the Baltic Sea be linked to internal nutrient levels and external nutrient load? Hydrobiologia. 2004;514:249–58.

[69] Kilham SS, Theriot EC, Fritz SC. Linking planktonic diatoms and climate change in the large lakes of the Yellowstone ecosystem using resource theory. Limnol Oceanogr. 1996;41(5):1052–62.

[70] Hughes L. Biological consequences of global warming: Is the signal already. Trends Ecol Evol. 2000;15(2):56–61. 10652556 10.1016/s0169-5347(99)01764-4

[71] Perrow MR, Meijer M, Dawidowicz P, Coops H. Biomanipulation in shallow lakes: State of the art. Hydrobiologia. 1997;342:355–65.

[72] Kirk JTO. Light and Photosynthesis in Aquatic Ecosystems. 3rd ed. Cambridge, UK: Cambridge University Press; 2011. 662 p.

[73] Harvey ET, Kratzer S, Andersson A. Relationships between colored dissolved organic matter and dissolved organic carbon in different coastal gradients of the Baltic Sea. Ambio. 2015;44(3):392–401. 26022322 10.1007/s13280-015-0658-4 PMC4447701

[74] Bricaud A, Morel A, Prieur L. Absorption by dissolved organic matter of the sea (yellow substance) in the UV and visible domains. Limnol Oceanogr. 1981;26(1):43–53.

[75] Kratzer S, Tett P. Using bio-optics to investigate the extent of coastal waters: A Swedish case study. Hydrobiologia. 2009;629(1):169–86.

[76] Sandel B, Smith AB. Scale as a lurking factor: Incorporating scale-dependence in experimental ecology. Oikos. 2009;118(9):1284–91.

[77] Donadi S, Austin ÅN, Bergström U, Eriksson BK, Hansen JP, Jacobson P, et al Cross-scale trophic cascade from large predatory fish to algae in coastal ecosystems. Proc R Soc London B Biol Sci [Internet]. 2017; Available from: http://dx.doi.org/10.1098/rspb.2017.0045 10.1098/rspb.2017.0045 PMC5543209 28724727

