# Supplementary material for: Relationships between aquatic vegetation and water turbidity: A field survey across seasons and spatial scales
Source: PLoS One. 2017 Aug 30;12(8):e0181419. doi: 10.1371/journal.pone.0181419 (PMC5576641; doi:10.1371/journal.pone.0181419)
Supplement: S1 Text — (PDF) [file pone.0181419.s001.pdf]

S1 Text. Description of the gillnet fishing in spring and how the relationship between turbidity and benthivorous fish was tested.

To control for sediment re-suspension by benthivorous fish, four to five Nordic survey gillnets (European Union standardized method EN 14757:2005) were set out in each bay overnight (from 4-7 pm to 7-10 am) in spring. This study was made in accordance to the ethical regulation laid down in the Swedish ordinance SJVFS 2012:26, which is the Swedish implementation of the Directive 2010/63/EU of the European Parliament and of the Council on the protection of animals used for scientific purposes. The fish died in the process of lifting the nets. All fish caught were identified to species, measured (total length in cm) and counted. Individual biomass was calculated using species-specific length:weight conversion factors (Swedish national database for coastal fish, http://www.slu.se/kul). Catch per unit effort (CPUE) was calculated as biomass (kg) per net and night of all fish species caught (S1 Table). In line with previous studies [45, 46], the density of benthivorous fish was calculated by dividing the total CPUE of black goby, bream, crucian carp, European flounder, ide, roach, rudd and tench by the area of each bay in km^2 (www.fishbase.org, accessed 2017-04-17). To test the effect of the density of benthivorous fish on turbidity we used a simple linear regression. There was no effect of the density of benthivorous fish on turbidity. We also tested the effect of salinity and topographic openness on the density of benthivorous fish. There was no effect of salinity, but a negative effect of topographic openness on the density of benthivorous fish (spring regional scale: estimate -0.90±0.27, $p < 0.01$, adjusted $R^2 = 0.24$).
