# Supplementary material for: Relationships between aquatic vegetation and water turbidity: A field survey across seasons and spatial scales
Source: PLoS One. 2017 Aug 30;12(8):e0181419. doi: 10.1371/journal.pone.0181419 (PMC5576641; doi:10.1371/journal.pone.0181419)
Supplement: S1 Table — The species are sorted after the highest average CPUE. Benthivorous fish species are marked in bold. (PDF) [file pone.0181419.s002.pdf]

S1 Table. Average CPUE over all bays of the fish species caught in gillnets in spring. The species are sorted after the highest average CPUE. Benthivorous fish species are marked in bold.

Common name	Scientific name	Average CPUE (kg/net and night)	Standard deviation
Perch	*Perca fluviatilis*	1.62	0.33
**Roach**	***Rutilus rutilus***	**0.91**	**0.23**
Pike	*Esox lucius*	0.55	0.11
**Bream**	***Abramis brama***	**0.25**	**0.20**
Three-spined stickleback	*Gasterosteus aculeatus*	0.23	0.045
Common bleak	*Alburnus alburnus*	0.19	0.11
Ruffe	*Gymnocephalus cernuus*	0.15	0.084
**Tench**	***Tinca tinca***	**0.13**	**0.43**
**Ide**	***Leuciscus idus***	**0.059**	**0.042**
Herring	*Clupea harengus*	0.056	0.21
**Rudd**	***Scardinius erythrophthalmus***	**0.018**	**0.13**
Sea trout	*Salmo trutta*	0.015	0.29
European smelt	*Osmerus eperlanus*	0.014	0.12
Shorthorn sculpin	*Myoxocephalus scorpius*	0.013	0.082
**Crucian carp**	***Carassius carassius***	**0.0097**	**0.17**
Nine-spined stickleback	*Pungitius pungitius*	0.0025	0.14
Eelpout	*Zoarces viviparus*	0.0024	0.14
Sprat	*Sprattus sprattus*	0.0021	0.20
Pike-perch	*Sander lucioperca*	0.0012	0.072
Great sandeel	*Hyperoplus lanceolatus*	0.00092	0.16
**Black goby**	***Gobius niger***	**0.00036**	**0.082**
**European flounder**	***Platichthys flesus***	**0.00021**	**0.0027**
Straightnose pipefish	*Nerophis ophidion*	0.000071	0.23
Broadnose pipefish	*Syngnathus typhle*	0.000054	2.0
Fifteen-spined stickleback	*Spinachia spinachia*	0.000047	0.068
