# Supplementary material for: Relationships between aquatic vegetation and water turbidity: A field survey across seasons and spatial scales
Source: PLoS One. 2017 Aug 30;12(8):e0181419. doi: 10.1371/journal.pone.0181419 (PMC5576641; doi:10.1371/journal.pone.0181419)
Supplement: S2 Table — The table shows the likelihood ratio test statistic (LRT), its p-value, the estimate and standard error of the fixed effect “Season” (level: summer) of the mixed models, for all variables at regional scale (n = 32) and local scale (N = 201). (PDF) [file pone.0181419.s003.pdf]

S2 Table. Results from the analyses of seasonal differences in variables.

Variable	Scale	LRT	p-value	Estimate	SE
Vegetation cover	Regional	7.00	0.0081	1.06	±0.39
	Local	23.40	<0.0001	1.22	±0.25
Turbidity	Regional	2.00	0.1577		
	Local	8.73	0.0031	0.05	±0.02
Fluorescence	Regional	6.18	0.0129	0.05	±0.02
	Local	48.14	<0.0001	0.05	±0.01
Sediement-driven turbidity	Regional	2.84×10^{-14}	1		
	Local	4.32×10^{-12}	1		
Salinity	Regional	13.32	<0.0001	0.29	±0.07
	Local	121.27	<0.0001	0.26	±0.02

The table shows the likelihood ratio test statistic (LRT), its p-value, the estimate and standard error of the fixed effect "Season" (level: summer) of the mixed models, for all variables at regional scale (N = 32) and local scale (N = 202).
