# Supplementary material for: Relationships between aquatic vegetation and water turbidity: A field survey across seasons and spatial scales
Source: PLoS One. 2017 Aug 30;12(8):e0181419. doi: 10.1371/journal.pone.0181419 (PMC5576641; doi:10.1371/journal.pone.0181419)
Supplement: S3 Table — At regional scale averages/bay were used. Bold numbers indicate the best fitting simplified models. Models that differ with less than 4 units of AIC are considered to fit the data equally well. (PDF) [file pone.0181419.s004.pdf]

S3 Table. Results from the directional separation test for model 1–4 at local and regional scale.

Variable of interest	Scale	Model	p-value	AIC
Turbidity	Regional	1	0.347	**43.88**
Turbidity	Regional	2	0.251	**45.59**
Turbidity	Regional	3	0.194	55.72
Turbidity	Regional	4	0.341	54.35
Turbidity	Local	1	0.331	**50.30**
Turbidity	Local	2	0.41	**48.86**
Turbidity	Local	3	0.41	**48.86**
Turbidity	Local	4	0.331	**50.30**
Fluorescence	Regional	1	0.931	**31.08**
Fluorescence	Regional	2	0.931	**31.08**
Fluorescence	Regional	3	0.931	**31.08**
Fluorescence	Regional	4	0.931	**31.08**
Fluorescence	Local	1	0.913	**37.67**
Fluorescence	Local	2	0.913	**37.67**
Fluorescence	Local	3	0.913	**37.67**
Fluorescence	Local	4	0.913	**37.67**
Sediment-driven turbidity	Regional	1	0.433	42.41
Sediment-driven turbidity	Regional	2	0.228	44.33
Sediment-driven turbidity	Regional	3	0.719	**35.96**
Sediment-driven turbidity	Regional	4	0.891	**33.07**
Sediment-driven turbidity	Local	1	0.576	**16.55**
Sediment-driven turbidity	Local	2	0.952	**12.70**
Sediment-driven turbidity	Local	3	0.952	**12.70**
Sediment-driven turbidity	Local	4	0.576	**16.55**

At regional scale averages/bay were used. Bold numbers indicate the best fitting simplified models. Models that differ with less than 4 units of AIC are considered to fit the data equally well.
