# Supplementary material for: Relationships between aquatic vegetation and water turbidity: A field survey across seasons and spatial scales
Source: PLoS One. 2017 Aug 30;12(8):e0181419. doi: 10.1371/journal.pone.0181419 (PMC5576641; doi:10.1371/journal.pone.0181419)
Supplement: S4 Table — Pearson correlation coefficients are shown below the diagonal, p-values are shown above the diagonal. Bold numbers show significant correlations. Topographic openness, retention time, nitrogen load, fluorescence and turbidity are log10-transformed, vegetation cover is square-root transformed. (PDF) [file pone.0181419.s005.pdf]

S4 Table. Correlation matrix for the regional scale data, with means per bay (n=32).

	**Topogr. open.**	**Retention time**	**Nitrogen load**	**Salinity spring**	**Salinity summer**	**Fluor. spring**	**Fluor. Summer**	**Turb. spring**	**Turb. summer**	**Sed.driv.turb spring**	**Sed.driv.turb summer**	**Veg.cover spring**	**Veg.cover summer**
**Topographic openness**	1.00	**0.00**	0.86	1.00	0.49	**0.04**	**0.03**	0.52	0.29	0.33	0.81	**0.03**	0.39
**Retention time**	**-0.92**	1.00	0.35	0.42	0.90	0.19	**0.05**	0.53	0.15	0.73	0.66	**0.01**	0.13
**Nitrogen load**	0.03	-0.17	1.00	0.06	**0.01**	0.07	0.07	**0.03**	**0.01**	0.25	0.07	0.58	0.97
**Salinity spring**	0.00	0.15	-0.34	1.00	**0.00**	0.31	0.53	0.12	0.37	0.40	0.56	0.57	0.79
**Salinity summer**	0.13	0.02	**-0.44**	**0.90**	1.00	0.17	0.22	**0.02**	**0.03**	0.11	0.12	0.86	0.72
**Fluorescence spring**	**-0.37**	0.24	0.32	-0.18	-0.25	1.00	**0.04**	**0.00**	0.23	0.95	0.98	0.33	0.76
**Fluorescence summer**	**-0.38**	**0.35**	0.32	-0.11	-0.22	**0.37**	1.00	0.51	**0.01**	0.81	0.94	0.18	0.42
**Turbidity spring**	-0.12	0.12	**0.38**	-0.28	**-0.41**	**0.57**	0.12	1.00	**0.02**	**0.00**	**0.03**	0.06	**0.04**
**Turbidity summer**	-0.19	0.26	**0.46**	-0.16	**-0.38**	0.22	**0.43**	**0.41**	1.00	**0.02**	**0.00**	0.07	**0.01**
**Sed.driv.turb spring**	0.18	-0.06	0.21	-0.15	-0.29	-0.01	-0.05	**0.76**	**0.40**	1.00	**0.00**	0.06	**0.00**
**Sed.driv.turb summer**	0.05	0.08	0.32	-0.11	-0.28	0.01	-0.01	**0.39**	**0.87**	**0.50**	1.00	0.10	**0.01**
**Veg.cover spring**	**0.39**	**-0.48**	0.10	-0.10	-0.03	-0.18	-0.24	-0.34	-0.32	-0.34	-0.29	1.00	**0.00**
**Veg.cover summer**	0.16	-0.27	-0.01	-0.05	0.07	-0.06	-0.15	**-0.37**	**-0.46**	**-0.54**	**-0.46**	**0.51**	1.00

Pearson correlation coefficients are shown below the diagonal, p-values are shown above the diagonal. Bold numbers show significant correlations.

Topographic openness, retention time, nitrogen load, fluorescence and turbidity are $\log_{10}$-transformed, vegetation cover is square-root transformed.
