# Supplementary figures and images for: Relationships between aquatic vegetation and water turbidity: A field survey across seasons and spatial scales
Source: PLoS One. 2017 Aug 30;12(8):e0181419. doi: 10.1371/journal.pone.0181419 (PMC5576641; doi:10.1371/journal.pone.0181419)

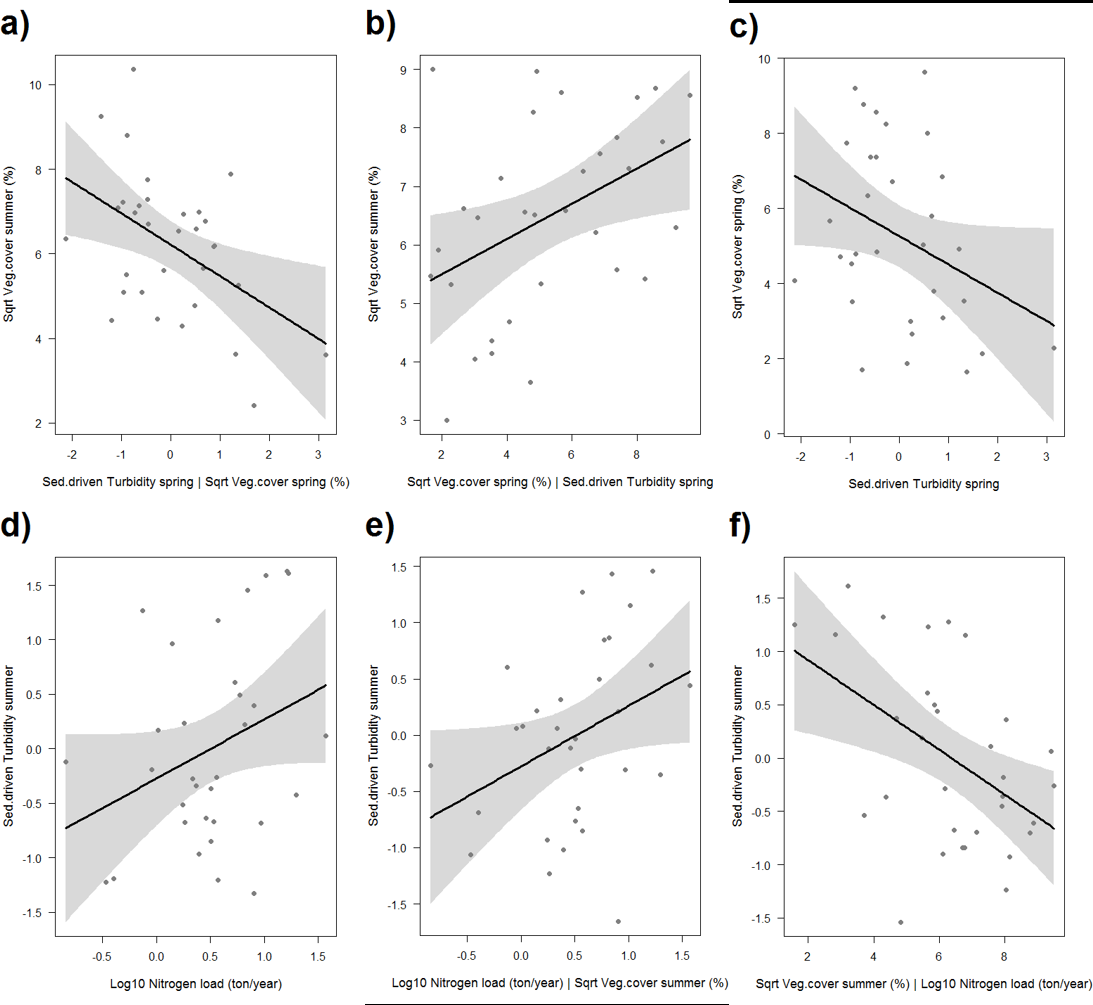

Supplement: S1 Fig — The sediment-driven turbidity is the residuals of turbidity predicted by fluorescence. The relationships shown are from the two best fitting models (model 3 and 4). Nitrogen load is log10-transformed, and vegetation cover is square-root transformed. (a)partial effect of sediment-driven turbidity in spring on vegetation cover in summer, given the effect of the co-variable vegetation cover in spring; (b) partial effect of vegetation cover in spring on vegetation cover in summer, given the effect of the co-variable sediment-driven turbidity in spring; (c) effect of sediment-driven turbidity in spring on vegetation cover in spring (p = 0.0563); (d) effect of nitrogen load on sediment-driven turbidity in summer in model 3 (p = 0.0730); (e) partial effect of nitrogen load on sediment-driven turbidity in summer, given the effect of the co-variable vegetation cover in summer in model 4; (f) partial effect of vegetation cover in summer on sediment-driven turbidity in summer, given the effect of the co-variable nitrogen load in model 4. (TIF) [file pone.0181419.s006.tif]
